# Synthesis and SAR Studies of Praziquantel Derivatives with Activity against *Schistosoma japonicum*

**DOI:** 10.3390/molecules18089163

**Published:** 2013-07-31

**Authors:** Wen-Long Wang, Li-Jun Song, Xia Chen, Xu-Ren Yin, Wen-Hua Fan, Gu-Ping Wang, Chuan-Xin Yu, Bainian Feng

**Affiliations:** 1 School of Pharmaceutical Science, Jiangnan University, Wuxi 214122, China; E-Mails: wenlongwang@jiangnan.edu.cn (W.-L.W.); chenxia19891219@126.com (X.C.); p85615822@yeah.net (G.-P.W.); 2 Jiangshu Alpha Biopharmaceuticals, Inc., Wuxi 214122, China; E-Mail: fanwenhua1987@163.com (W.-H.F.); 3 Key Laboratory on Technology for Parasitic Disease Prevention and Control, Ministry of Health, Jiangsu Institute of Parasitic Diseases, Wuxi 214064, China; E-Mails: songlijun1025@163.com (L.-J.S.); yinxuren@sohu.com (X.-R.Y.)

**Keywords:** praziquantel derivatives, antischistosomal activity, *Schistosoma japonicum*, neglected tropical disease

## Abstract

The synthesis and structure-activity relationship (SAR) studies of praziquantel derivatives with activity against adult *Schistosoma japonicum* are described. Several of them showed better worm killing activity than praziquantel and could serve as leads for further optimization.

## 1. Introduction

Schistosomiasis is a neglected tropical disease (NTD) caused by blood-dwelling trematodes belonging to the genus Schistosoma. *Schistosoma haematobium*, *Schistosoma japonicum*, and *Schistosoma mansoni* are the main species parasitizing humans [1]. It has been estimated that some 779 million people are at risk for schistosomiasis transmission, with 207 million infected in 76 countries and territories [1,2]. In China, the disease caused by *Schistosoma japonicum* remains a major public health concern with more than 280 thousand people infected [3]. In the absence of a vaccine, praziquantel (PZQ) has been the only drug recommended by the World Health Organization for the treatment and control of schistosomiasis through mass drug administration (MDA) programs for almost three decades [4]. However, PZQ does not prevent reinfection, and it is inactive against juvenile schistosomes. Furthermore, it has only a limited effect on developed liver and spleen lesions, as well as on the emergent schistosome phenotypes that are resistant to PZQ chemotherapy [5,6]. Therefore, it is imperative to develop new antischistosomal agents. A number of recent studies were directed towards exploration of new chemical entities from natural products [7,8,9,10] and towards the identification of additional drug targets for schistosomiasis [11,12,13,14,15].

In addition, there are two other major issues with regard to PZQ: (1) the mechanism of action of PZQ is uncertain [16] and (2) as currently administered, PZQ is a racemic mixture [16]. It is a great challenge to prepare (−)-PZQ inexpensively and stereoselectively [17]. The less active (+)-PZQ enantiomer has well been documented as having a bitter and disgusting taste [18]. Recently, many synthetic routes for PZQ and its derivatives have been developed [19,20], providing more opportunities to make novel PZQ derivatives as potential drugs for the treatment of schistosomiasis.

**Figure 1:**
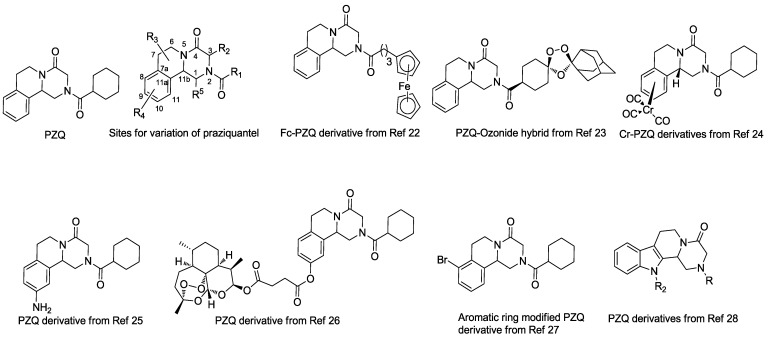
Chemical structure of praziquanel (PZQ) and some reported derivatives.

Ever since the discovery of PZQ, there has been interest in the elucidation of the structure–activity relationships (SAR) [21], mostly from data generated using *S.* *mansoni*, and to some extent with data for *S. japonicum*. There are five positions amenable for chemical modification in the PZQ molecular structure [16,22] (Figure 1). Among these, only position R_1_ has been heavily investigated [21]. Recently, more variants at the R_1_ position as well as PZQ-ozonide hybrids were synthesized and tested against juvenile and adult stages of *S. mansoni* *in vitro* and *in vivo*. Unfortunately, all the variants showed decreased activity compared to the parent compound PZQ [23]. Patra and co-workers reported PZQ analogues that replaced the cyclohexyl group at position 2 with different ferrocenyl moieties. Unfortunately, the Fc-PZQ derivatives were considerably less active than PZQ when tested against *S. mansoni in vitro* [22]. Recently, Gasser and coworkers reported Cr-PZQ derivatives with comparable antischistosomal activities to PZQ when tested against adult *S. mansoni* [24]. In 2007, the Todd group first reported several derivatives through nitration and amination of the C10 position of the aromatic ring on PZQ. However, these compounds showed significantly decreased worm killing activity against *S. mansoni in vitro* [25]. In 2012, the Qiao group synthesized eight PZQ/peroxide conjugates, with some compounds showing superior worm-killing activity against *S. japonicum* in *in vitro* assays compared to PZQ [26]. Recently, the Qiao group reported aromatic ring-modified praziquantel derivatives with activity against both juvenile and adult *S. japonicum*, and a compound with a bromide atom at C8 position showed higher potency against adult *S. japonicum* than PZQ *in vitro* and comparable antischistosomal activity to PZQ *in vivo*. However, none of them showed superior worm-killing ability to PZQ [27]. The Rao group developed an efficient synthetic route to provide structurally diverse analogues for SAR studies. Several PZQ analogues with an indol ring were synthesized and tested against adult *S. mansoni*. Unfortunately, no compounds showed better activity than PZQ *in vitro* [28]. Taken together, all this data indicates that although much work has been done on PZQ, it is still necessary to further expand the range of PZQ analogues in order to obtain chemical probes to elucidate the real biological mechanism of PZQ, and to generate alternative new chemotherapies. Therefore, we became interested in the synthesis of new PZQ analogues to find new potential antischistosomal agents against *S. japonicum*.

## 2. Results and Discussion

### 2.1. Chemistry

**Scheme 1:**
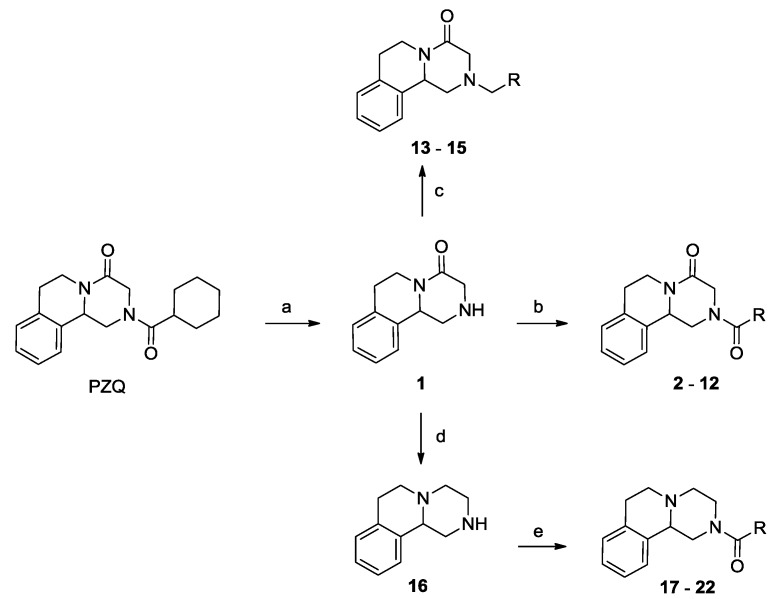
Synthesis of PZQ analogues **1**–**22**.

**Scheme 2:**
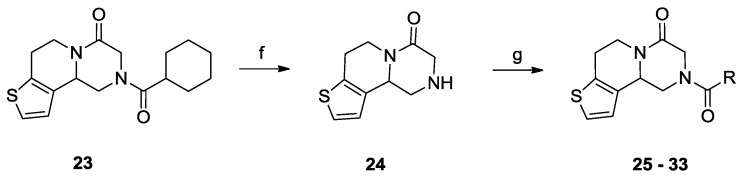
Synthesis of PZQ analogues **24**–**33**.

Due to the lack of SAR details from *S. japonicum*, we initially resynthesized some PZQ analogues with position 2 substitution and evaluated their worm-killing activities against *S. japonicum* (Scheme 1). The racemic PZQ from Aldrich was hydrolyzed by 2 N HCl to yield praziquanamine (**1**) using the method described by the Vennerstrom group [23], followed by coupling with acyl chlorides to yield compounds **2**–**12**. Reductive amination reactions of appropriate aldehydes with praziquanamine (**1**) were performed with sodium triacetoxyborohydride in dichloroethane at room temperature to afford compounds **13**–**15** in yields ranging from 27% to 35%. Praziquanamine (**1**) was reduced by lithium aluminum hydride to yield compound **16**, followed by acylation to yield compounds **17**–**22**. To extend the SAR studies, the aromatic moiety was replaced by a thiophene ring, and compound **23** was synthesized using the method described by Frehel and his coworkers [29]. Compound **23** was hydrolyzed with 2N HCl to yield amine **24**, followed by acylation with appropriate acyl chlorides to yield compounds **25**–**33** (Scheme 2). As in previous reports [22], we also observed the major isomer in mixtures with its minor enantiomer with respect to the 11b position on the ring system for compounds **1**–**33**. The results were also in agreement with the nature of the commercially available PZQ [19,30] and its thiophene analogue **23** [20].

*Reagents and conditions*: (a) 2 N HCl, reflux, overnight, 62%; (b) TFAA, Et_3_N, DCM, 62% or RCOCl, Et_3_N, DCM, 29%–50%; (c) RCHO, NaBH_4_, HOAc, MeOH, 0 °C to 60 °C, 27%–35%; (d) LiAlH_4_, THF, 0 °C, 35%; (e) RCOCl, Et_3_N, DCM, 32%–69%.

*Reagents and conditions*: (f) 2 N HCl, reflux, overnight, 78%; (g) RCOCl, Et_3_N, DCM, 40%–76%.

### 2.2. Biological Activities

The biological results are listed in Table 1. Among compounds with an aliphatic cyclic ring (**2**–**4** and PZQ), the worm-killing activity was improved by increasing the ring size. Compounds with an acetyl group (*i.e*., **5**) and with an isobutyryl group (*i.e*., **6**) lacked worm-killing activity. Among compounds with a halogen atom, compound **7** with the chloroacetyl group killed 100% of the worms at the concentration of 5 μM, showing better activity than PZQ. However, compound **8** with a 2,2,2-trifluoroacetyl group lost worm killing activity. Replacement of the cyclohexyl ring with a heterocyclic ring, (compound **11** with a thiophene-2-carbonyl group) led to a similar activity to that of PZQ. However, compound **10** with an isonicotinoyl group and compound **12** with a furan-2-carbonyl group did not show any worm-killing activity below the concentration of 100 μM. Compound **9**, with a benzoyl group, showed similar activity as PZQ. Replacement of the carbonyl group at position 2 with CH_2_ gave compounds **13**–**15**, which showed no worm-killing activity. The results indicated that the amide bond at position 2 is essential for activity against *S. japonicum*. The SAR results displayed similarities between compounds **17**–**22** and compounds containing amide (**3**, **4**, PZQ, **9**, **11**, and **7**), showing decreased activity. The results indicated that the amide bond at position 4 was very important to maintain the activity of PZQ derivatives. The SAR results from *S. japonicum* adult worms were similar with those from *S.* *mansoni* [21]. However, it still gave us valuable information to explore novel PZQ derivatives as antischistosomal agents. 

To enlarge the chemical space of potential drug candidates, replacement of the phenyl ring on PZQ with a thiophene ring gave compounds **23**–**33**. Among compounds with an alkyl group (*i.e*., **23**, **25**–**29**), compound **23** with a cyclohexanecarbonyl group showed the best worm-killing activity, and it showed similar activity to PZQ. However, the worm-killing activity of compounds **24**–**29** was dramatically decreased. The amine-containing compound **24** had no activity. Among compounds with an aromatic ring, compound **30** with a benzoyl group showed slight worm-killing activity, while compound **31** with a thiophene-2-carbonyl group and compound **32** with a furan-2-carbonyl group had no activity. Interestingly, compound **33** with a chloroacetyl group showed good worm killing activity at 10 μM, similar to PZQ derivative **7**. Among the compounds reported here, compounds **7**, **22** and **33** with a chloroacetyl group showed better antischistosomal activity than PZQ. The evaluation of their cytotoxity is in progress. There have been many compounds with a chloroacetyl group under development [31,32,33]. For example, TNP470 [*O*-(chloroacetylcarbamoyl)fumagillol], which was derived from the natural product fumagillin, was in clinical trials for Kaposi’s sarcoma [34], as well as renal, brain, breast, cervical and prostate cancers [35].

**Table 1:** Worm-killing activity on *S. japonicum* adult worms *in vitro* by compounds **1**–**33**. 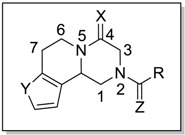

Compound	R	X	Y	Z	Killing activity ^a^			
					Conc (μM) ^b^	24 h	48 h	72 h
Vehicle						n.e	n.e	n.e
PZQ					10	25.0%D	25.0%D	37.5%D
					25	25.0%D	25.0%D	62.5%D
					50	25.0%D	37.5%D	62.5%D
					100	37.5%D	50.0%D	87.5%D
**1** ^d, e^	None	O	CH=CH	None	100	n.e.	n.e.	n.e.
**2** ^d, e^	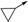	O	CH=CH	O	100	sluggish	25.0%	75.0%
**3** ^e^	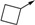	O	CH=CH	O	10	n.e.	n.e.	sluggish
					25	n.e.	n.e.	10.0%D
					50	n.e.	sluggish	75.0%D
					100	sluggish	sluggish	87.5%D
**4** ^e^	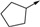	O	CH=CH	O	10	sluggish	sluggish	37.5%D
					25	sluggish	sluggish	50.0%D
					50	sluggish	sluggish	100%D
					100	12.5%D	12.5%D	75.0%D
**5** ^d, e^	CH_3_	O	CH=CH	O	100	n.e.	n.e.	n.e.
**6** ^d, e^	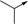	O	CH=CH	O	100	n.e.	n.e.	20.0%D
**7** ^c, e^	ClCH_2_	O	CH=CH	O	1	n.e.	n.e.	n.e.
					3	n.e.	25.0%D	25.0%D
					5	70.0%D	90.0%D	100%D
					8	100%D	100%D	100%D
					10	100%D	100%D	100%D
					25	100%D	100%D	100%D
					50	100%D	100%D	100%D
					100	100%D	100%D	100%D
**8** ^d, e^	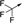	O	CH=CH	O	100	n.e.	n.e.	n.e.
**9** ^e^	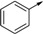	O	CH=CH	O	10	n.e.	n.e.	55.6%D
					25	sluggish	25.0%D	87.5%D
					50	18.2%D	36.4%D	45.5%D
					100	45.5%D	45.5%D	81.8%D
**10** ^d, e^	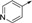	O	CH=CH	O	100	n.e.	n.e.	sluggish
**11** ^e^	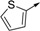	O	CH=CH	O	10	n.e.	n.e.	62.5%D
					25	n.e.	n.e.	62.5%D
					50	25.0%D	37.5%D	87.5%D
					100	14.3%D	28.6%D	87.5%D
**12** ^d, e^	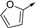	O	CH=CH	O	100	n.e.	n.e.	n.e.
**13** ^d^	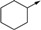	O	CH=CH	H_2_	100	n.e.	n.e.	n.e.
**14** ^d, e^	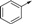	O	CH=CH	H_2_	100	n.e.	n.e.	n.e.
**15** ^d^	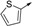	O	CH=CH	H_2_	100	n.e.	n.e.	n.e.
**16** ^d, e^	None	H_2_	CH=CH	None	100	n.e.	n.e.	n.e.
**17** ^d^	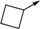	H_2_	CH=CH	O	100	n.e.	n.e.	n.e.
**18**	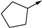	H_2_	CH=CH	O	10	sluggish	sluggish	sluggish
					25	sluggish	18.2%D	18.2%D
					50	10.0%D	20.0%D	50.0%D
					100	25.0%D	25.0%D	75.0%D
**19**	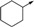	H_2_	CH=CH	O	10	n.e.	n.e.	n.e.
					25	n.e.	14.3%D	57.1%D
					50	sluggih	33.3%D	66.7%D
					100	sluggish	sluggish	85.7%D
**20** ^d, e^	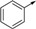	H_2_	CH=CH	O	100	n.e.	n.e.	n.e.
**21** ^d^	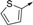	H_2_	CH=CH	O	100	n.e.	n.e.	n.e.
**22**	ClCH_2_	H_2_	CH=CH	O	10	n.e.	sluggish	50.0%D
					25	87.5%D	100%D	100%D
					50	100%D	100%D	100%D
					100	100%D	100%D	100%D
**23** ^e^	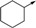	O	S	O	10	sluggish	75.0%D	75.0%D
					25	sluggish	87.5%D	87.5%D
					50	25.0%	75.0%D	87.5%D
					100	28.6%D	71.4%D	71.4%D
**24** ^d^	None	O	S	None	100	n.e.	n.e.	n.e.
**25** ^d^	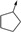	O	S	O	100	n.e.	12.5%D	37.5%D
**26** ^d^	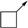	O	S	O	100	n.e.	sluggish	sluggish
**27** ^d^	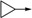	O	S	O	100	n.e.	n.e.	sluggish
**28** ^d^	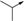	O	S	O	100	n.e.	n.e.	n.e.
**29** ^d^	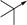	O	S	O	100	sluggish	sluggish	sluggish
**30** ^e^	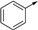	O	S	O	10	n.e.	n.e.	n.e.
					25	n.e.	sluggish	sluggish
					50	n.e.	14.3%D	14.3%D
					100	sluggish	37.5%D	75.0%D
**31** ^d, e^	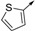	O	S	O	100	n.e.	n.e.	n.e.
**32** ^d, e^	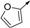	O	S	O	100	n.e.	n.e.	n.e.
**33** ^c^	ClCH_2_	O	S	O	1	n.e.	n.e.	n.e.
					3	n.e.	n.e.	n.e.
					5	sluggish	25%D	37.5%D
					8	75%D	87.5%D	87.5%D
					10	100%D	100%D	100%D
					25	100%D	100%D	100%D
					50	100%D	100%D	100%D
					100	100%D	100%D	100%D

^a^ Data collected by visual examination of worm movement and shape (the values were the averages of two tests); n.e. (no effect): all worms are scored as active in culture with typical appearance; sluggish: worm movement is significantly reduced; % D = number of dead worms/total number of worms observed, and dead worms judged by lack of movement within 2 minutes in addition to morphological and tegumental alterations; ^b^ The concentration of the chemicals on *S. japonicum* adult worms *in vitro*; ^c^ Starting concentration of 1 μM when the chemicals killed 100% of the worms at the concentration of 10 μM; ^d^ Data only showed the concentration of 100 μM when the chemicals have no effect on the worms at 10, 25 and 50 μM; ^e^ Reported in References [20,21,29] and references therein.

## 3. Experimental

### 3.1. Chemistry

All chemicals were reagent grade and used as purchased. All reactions were performed under an inert atmosphere of dry argon or nitrogen using distilled dry solvent. ^1^H-NMR spectra (400 MHz) were recorded on a Bruker AVⅢ 400 MHz spectrometer. The chemical shifts were reported in (ppm) using the 7.26 signal of CDCl_3_ (^1^H-NMR) as internal standards. Mass spectra (MS) were obtained on a Waters Micromass Platform LCZ Mass Spectrometer.

#### 3.1.1. Procedure for the Preparation of Compound **1**

A mixture of PZQ (10 g, 32 mmol) and 2 N HCl (50 mL) was refluxed overnight. After PZQ was consumed, the reaction solution was cooled to room temperature, neutralized with NaHCO_3_ (aq.) and then extracted with DCM/MeOH (V/V 10/1, 100 mL × 3). The organic layer was washed with water and brine, dried with anhydrous Na_2_SO_4_ and concentrated to get the crude product. The crude product was washed with petroleum ether/EtOAc (V/V 10/1) to yield compound **1** (4 g, 62%) as a yellow solid. ^1^H-NMR (CDCl_3_) *δ*: 2.73–3.03 (m, 4H), 3.48–3.77 (m, 3H), 4.79–4.89 (m, 2H), 7.13–7.27 (m, 4H); MS (ESI): *m/z* calcd for C_12_H_15_N_2_O [M+H]^+^: 203.1, found: 203.4.

#### 3.1.2. Procedure for the Preparation of Compound **8**

A stirred solution of compound **1** (500 mg, 2.5 mmol) in DCM (20 mL) was added to trifluoroacetic anhydride (275 μL, 2 mmol) and triethylamine (275 μL, 2 mmol). After the addition, the mixture was stirred at room temperature overnight. The reaction residue was poured into water, and extracted with EtOAc (50 mL × 3). The organic phases were then processed in the usual way and chromatographed (1:1 petroleum ether/EtOAc) to afford compound **8** (450 mg, 62%) as a white solid. ^1^H-NMR (CDCl_3_) δ:2.96 (m), 3.38 (dd, *J* = 10.8 Hz, 14.0 Hz) (total 4H), 4.00 (d, *J* = 18.4 Hz), 4.26 (d, *J* = 17.6 Hz) (total 1H), 4.61 (dd, *J* = 14.0Hz, 18.0 Hz, 1H), 4.84-5.10 (m, 3H), 7.16–7.34 (m, 4H); MS (ESI): *m/z* calcd. for [M+H]^+^: 299.1, found: 299.2.

#### 3.1.3. General Procedure for the Preparation of Derivatives **2**–**7** and **9**–**12**

A stirred solution of compound **1** (500 mg, 2.5 mmol) in DCM (50 mL) was added to cyclopropanecarbonyl chloride (420 μL, 3.7 mmol) at 0 °C slowly. After the addition, the mixture was stirred at room temperature overnight. The reaction residue was poured into NaHCO_3_ (aq.), extracted with DCM (250 mL), washed with water and brine/dried over Na_2_SO_4_. The organic phases were then processed in the usual way and chromatographed (1:1 petroleum ether/EtOAc) to afforded compound **2** (350 mg, 49%). ^1^H-NMR (CDCl_3_) *δ*: 0.89 (m, 2H), 1.04-1.15 (m, 2H), 1.73 (m, 1H), 2.79–3.02 (m, 4H), 4.23 (d, *J* = 17.2 Hz, 1H), 4.71 (d, *J* = 17.6 Hz, 1H), 4.84-4.87 (m, 2H), 5.15 (d, *J* = 12.8 Hz, 1H), 7.20–7.28 (m, 4H); MS (ESI): *m/z* calcd. for [M+H]^+^: 271.1, found: 271.2. The following compounds were similarly prepared.

*2-(Cyclobutanecarbonyl)-2,3,6,7-tetrahydro-1H-pyrazino[2,1-a]isoquinolin-4(11bH)-one* (**3**)*.* Yield = 40%; ^1^H-NMR (CDCl_3_) *δ*: 1.88-2.11 (m, 2H), 2.19–2.27 (m, 2H), 2.32–2.50 (m, 2H), 2.78–3.03 (m, 4H), 3.15–3.42 (m, 1H), 3.85 (d, *J* = 18.8 Hz), 3.99 (d, *J* = 17.2 Hz) (total 1H), 4.27 (d, *J* = 17.6 Hz, 1H), 4.74–4.91 (m, 2H), 5.14 (m, 1H), 7.17–7.33 (m, 4H); MS (ESI): *m/z* calcd. for [M+H]^+^: 285.2, found: 285.3.

*2-(Cyclopentanecarbonyl)-2,3,6,7-tetrahydro-1H-pyrazino[2,1-a]isoquinolin-4(11bH)-one* (**4**)*.* Yield = 43%; ^1^H-NMR (CDCl_3_) *δ*: 1.62–1.96 (m, 8H), 2.78–3.02 (m, 5H), 3.54–3.90 (m, 1H), 4.10 (d, *J* = 17.2 Hz), 4.51 (d, *J* = 17.2 Hz) (total 1H), 4.82–4.4.91 (m), 5.18 (dd, *J* = 2.8 Hz, 13.2 Hz)(total 3H), 7.16–7.30 (m, 4H); MS (ESI): *m/z* calcd. for [M+H]^+^: 299.2, found: 299.4 [M+H]^+^.

*2-Acetyl-2,3,6,7-tetrahydro-1H-pyrazino[2,1-a]isoquinolin-4(11bH)-one* (**5**)*.* Yield = 43%; ^1^H-NMR (CDCl_3_) *δ*: 2.19 (s), 2.25 (s) (total 3H), 2.79–3.01 (m), 3.29–3.35 (m) (total 4H), 3.92 (d, *J* = 18.4 Hz), 4.11 (d, *J* = 17.6 Hz) (total 1H), 4.31–4.47 (m), 4.38 (d, *J* = 17.6 Hz) (total 1H), 4.77–4.89 (m, 2H), 5.13–5.18 (m, 1H), 7.20–7.33 (m, 4H); MS (ESI): *m/z* calcd. for [M+H]^+^: 245.1, found: 245.1.

*2-Isobutyryl-2,3,6,7-tetrahydro-1H-pyrazino[2,1-a]isoquinolin-4(11bH)-one* (**6**). Yield = 43%; ^1^H- NMR (CDCl_3_) *δ*: 1.10 (d, *J* = 6.8 Hz), 1.13 (d, *J* = 6.8Hz) (total 6H), 2.71–2.97 (m), 3.21 (m) (total 5H), 3.81 (d, *J* = 18.4 Hz), 4.01–4.09 (m) (total 1H), 4.41(d, *J* = 17.2 Hz, 1H), 4.76 (m, 2H), 5.11 (dd, *J* = 2.4 Hz, 12.8 Hz, 1H), 7.12–7.23 (m, 4H); MS (ESI): *m/z* calcd. for [M+H]^+^: 273.2, found: 273.0.

*2-(2-Chloroacetyl)-2,3,6,7-tetrahydro-1H-pyrazino[2,1-a]isoquinolin-4(11bH)-one* (**7**). Yield = 44%; ^1^H-NMR (CDCl_3_) *δ*: 2.78–2.96 (m), 3.28–3.34 (m) (total 4H), 3.89 (d, *J* = 18.4 Hz), 4.19–4.26 (m) (total 1H), 4.19 (s), 4.15 (s) (total 2H), 4.42–4.46 (m, 1H), 4.77–4.88 (m, 2H), 5.02–5.08 (m, 1H), 7.19–7.28 (m, 4H); MS (ESI): *m/z* calcd. for [M+H]^+^: 279.1/281.1, found: 279.2/281.3.

*2-Benzoyl-2,3,6,7-tetrahydro-1H-pyrazino[2,1-a]isoquinolin-4(11bH)-one* (**9**)*. * Yield = 34%; ^1^H-NMR (CDCl_3_) *δ*: 2.77–3.10 (m, 4H), 4.06–4.11 (m, 1H), 4.25–4.39 (m, 1H), 4.82–4.99 (m, 2H), 5.25 (m, 1H), 7.19–7.49 (m, 9H); MS (ESI): *m/z* calcd for [M+H]^+^: 307.1, found: 307.2.

*2-Isonicotinoyl-2,3,6,7-tetrahydro-1H-pyrazino[2,1-a]isoquinolin-4(11bH)-one* (**10**)*. * Yield = 29%; ^1^H-NMR (CDCl_3_) *δ*: 2.80–3.30 (m, 4H), 4.10–4.27 (m, 2H), 4.81–5.24 (m, 3H), 6.75 (m), 7.23–7.39 (m) (total 6H), 8.79 (2H); MS (ESI): *m/z* calcd. for [M+H]^+^: 308.1, found: 308.4.

*2-(Thiophene-2-carbonyl)-2,3,6,7-tetrahydro-1H-pyrazino[2,1-a]isoquinolin-4(11bH)-one* (**11**)*. * Yield = 29%; ^1^H-NMR (CDCl_3_) *δ*: 2.80–2.85 (m, 1H), 2.90–3.04 (m, 2H), 3.12–3.18 (m, 1H), 4.27 (d, *J* = 17.6 Hz, 1H), 4.86 (m, 2H), 5.03 (dd, *J* = 4.0 Hz, 10.8 Hz, 1H), 5.11 (d, *J* = 11.2 Hz, 1H), 7.14 (dd, *J* = 3.6 Hz, 4.8 Hz, 1H), 7.22–7.30 (m, 4H), 7.46 (d, *J* = 3.6 Hz, 1H), 7.57 (d, *J* = 4.4 Hz, 1H); MS (ESI): *m/z* calcd. for [M+H]^+^: 313.1, found: 313.2.

*2-(Furan-2-carbonyl)-2,3,6,7-tetrahydro-1H-pyrazino[2,1-a]isoquinolin-4(11bH)-one* (**12**). Yield = 50%; ^1^H-NMR (CDCl_3_) *δ*: 2.80–3.19 (m, 4H), 4.29 (m, 1H), 4.87 (d, *J* = 11.2 Hz, 1H), 5.01 (d, *J* = 15.2 Hz, 2H), 5.19 (d, *J* = 11.2 Hz, 1H), 6.56 (d, *J* = 3.2 Hz, 1H), 7.19 (d, *J* = 3.6 Hz, 1H), 7.22–7.31 (m, 4H), 7.58 (s, 1H); MS (ESI): *m/z* calcd. for [M+H]^+^: 297.1, found: 297.2.

#### 3.1.4. General Procedure for the Preparation of Derivatives **13**–**15**

To a solution of compound **1** (305 mg, 1.5 mmol) in methanol (10 mL), cyclohexanecarbaldehyde (186 μL, 1.5 mmol) was added at 0 °C, followed by acetic acid (170 μL, 3.0 mmol) addition. The mixture was thus maintained at 0 °C for 1 h. Then it was heated at 60 °C for 2 h. After cooled to 0 °C, NaBH_4_ (0.45 g, 12.0 mmol) was added by portions. The reaction mixture was stirred at 60–70 °C for 12 h, followed by evaporation to remove methanol. The residue was diluted with water (30 mL) and extracted with ethyl acetate (30 mL × 3). The organic phases were then processed in the usual way and chromatographed (2:1 petroleum ether/EtOAc) to afforded compound **13** (140 mg, 32%) as a white solid. ^1^H-NMR (CDCl_3_) *δ*: 0.89–0.95 (m, 4H), 1.59–1.83 (m, 6H), 2.23–2.35 (m, 3H), 2.75–2.99 (m, 4H), 3.46–3.60 (m, 3H), 4.80–4.90 (m, 2H), 7.16–7.28 (m, 4H); MS (ESI): *m/z* calcd. for [M+H]^+^: 299.2, found: 299.3. The following compounds were similarly prepared.

*2-Benzyl-2,3,6,7-tetrahydro-1H-pyrazino[2,1-a]isoquinolin-4(11bH)-one* (**14**)*.* Yield = 35%; ^1^H-NMR (CDCl_3_) *δ*: 2.37 (t, *J* = 10.8 Hz, 1H), 2.75 (d, *J* = 14.8 Hz, 1H), 2.86–2.97 (m, 3H), 3.49–3.59 (m, 2H), 3.64 (AB, *J* = 13.2 Hz, 2H), 4.78 (m, 1H), 4.87 (m, 1H), 6.97-7.36 (m, 9H); MS (ESI): *m/z* calcd. for [M+H]^+^: 293.2, found: 293.4. 

*2-(Thiophen-2-ylmethyl)-2,3,6,7-tetrahydro-1H-pyrazino[2,1-a]isoquinolin-4(11bH)-one* (**15**)*.* Yield = 27%; ^1^H-NMR (CDCl_3_) *δ*: 2.37 (t, *J* = 10.4 Hz, 1H), 2.75 (d, *J* = 14.8 Hz, 1H), 2.89–3.02 (m, 3H), 3.49–3.63 (m, 2H), 3.86 (s, 2H), 4.79 (m, 1H), 4.89(m, 1H), 6.97–7.29 (m, 7H). MS (ESI): *m/z* calcd. for [M+H]^+^: 299.1, found: 299.0.

#### 3.1.5. Procedure for the Preparation of Compound **16**

Compound **1** (3.03 g, 15 mmol) was added to a solution of lithium aluminum hydride (1.14 g, 30 mmol) in tetrahydrofuran (63 mL) and the mixture was refluxed for 10 h After cooled to 0 °C, the reaction was quenched with aqueous sodium hydroxide (15%, 1.2 mL), and the precipitate was filtrated. The organic phases were then processed in the usual way and chromatographed (10:1 CH_2_Cl_2_/MeOH) to afforded compound **16** (1 g, 35%) as red oil. ^1^H-NMR (CDCl_3_) *δ*: 2.39–2.56 (m, 2H), 2.62–2.68 (m, 2H), 2.83–2.91 (m, 2H), 2.97 (dd, *J* = 2.8 Hz, 8.4 Hz, 2H), 3.07–3.15 (m, 1H), 3.25 (d, *J* = 10.4 Hz, 1H), 3.52 (dd, *J* = 2.8 Hz, 12.0 Hz, 1H), 4.58 (brs, 1H), 7.04–7.13 (m, 4H); MS (ESI): *m/z* calcd. for [M+H]^+^: 189.1, found:189.3.

#### 3.1.6. General Procedure for the Preparation of Derivatives **17**–**22**

To a stirred solution of compound **16** (500 mg, 2.7 mmol) in DCM (50 mL), cyclohexanecarbonyl chloride (532 μL, 4.0 mmol) was added at 0 °C. The mixture was stirred at room temperature overnight. The reaction was quenched with NaHCO_3_ (aq.), extracted with DCM (50 mL × 3). The organic phases were then processed in the usual way and chromatographed (1:1 petroleum ether/ EtOAc) to afforded compound **19** (350 mg, 43%) as white solid. ^1^H-NMR (CDCl_3_) *δ*:1.24–1.87 (10H), 2.50 (m, 1H), 2.65 (m, 2H), 2.78 (m, 1H), 2.90–3.13 (m, 3H), 3.28 (m, 2H), 3.44 (m, 1H), 3.92 (d, *J* = 12.4 Hz), 4.37 (d, *J* =12.8 Hz) (total 1H), 4.65 (d, *J* = 13.2 Hz), 5.19 (d, *J* = 12.4 Hz) (total 1H), 7.13–7.33 (4H); MS (ESI): *m/z* calcd. for [M+H]^+^: 299.2, found: 299.1. The following compounds were similarly prepared.

*Cyclobutyl(3,4,6,7-tetrahydro-1H-pyrazino[2,1-a]isoquinolin-2(11bH)-yl)methanone* (**17**). Yield = 35%; ^1^H-NMR (CDCl_3_) *δ*: 1.92–2.01 (m, 2H), 2.15–2.25 (m, 2H), 2.33–2.50 (m, 3H), 2.54–2.64 (m, 2H), 2.75 (m, 1H), 2.87–3.04 (m, 3H), 3.15–3.46 (m, 3H), 3.68 (d, *J* = 12.8 Hz), 4.11-4.18 (m) (total 1H), 4.60 (d, *J* = 12.0 Hz), 5.16 (d, *J* = 12.4 Hz) (total 1H), 7.14–7.33 (m, 4H); MS (ESI): *m/z* calcd. for [M+H]^+^: 271.2, found: 271.3.

*Cyclopentyl(3,4,6,7-tetrahydro-1H-pyrazino[2,1-a]isoquinolin-2(11bH)-yl)methanone* (**18**)*. * Yield = 32%; ^1^H-NMR (CDCl_3_) *δ*: 1.58–1.98 (8H), 2.46 (m, 1H), 2.61 (m, 2H), 2.76 (d, *J* = 17.2 Hz, 1H), 2.91–3.10 (m, 3H), 3.23 (m, 2H), 3.40 (m, 1H), 3.97 (d, *J* = 12.4 Hz), 4.45 (d, *J* = 12.8 Hz) (total 1H), 4.66 (d, *J* = 12.8 Hz), 5.21 (d, *J* = 12.8 Hz) (total 1H), 7.13–7.33 (m, 4H); MS (ESI): *m/z* calcd. for [M+H]^+^: 285.2, found: 285.1.

*Phenyl(3,4,6,7-tetrahydro-1H-pyrazino[2,1-a]isoquinolin-2(11bH)-yl)methanone* (**20**)*. * Yield = 69%; ^1^H-NMR (CDCl_3_) *δ*: 2.40–2.60 (m, 2H), 2.66–2.71 (m, 1H), 2.79 (m, 1H), 2.97 (m, 2H), 3.13–3.33 (m, 3H), 3.67 (m), 4.18 (m) (total 1H), 4.64 (m), 5.21 (m) (total 1H), 6.58–7.38 (9H); MS (ESI): *m/z* calcd. for [M+H]^+^: 293.2, found: 293.2.

*(3,4,6,7-Tetrahydro-1H-pyrazino[2,1-a]isoquinolin-2(11bH)-yl)(thiophen-2-yl)methanone* (**21**)*. * Yield = 44%; ^1^H-NMR (CDCl_3_) *δ*: 2.61 (m, 2H), 2.77 (m, 1H), 3.00–3.08 (m, 3H), 3.18–3.43 (m, 3H), 4.44 (brs, 1H), 5.04 (brs, 1H), 7.12 (t, *J* = 4.4 Hz, 1H), 7.15–7.21 (m, 4H), 7.40 (d, *J* = 2.8 Hz, 1H), 7.51 (d, *J* = 5.2 Hz, 1H); MS (ESI): *m/z* calcd. for [M+H]^+^: 299.1, found: 299.3.

*2-Chloro-1-(3,4,6,7-tetrahydro-1H-pyrazino[2,1-a]isoquinolin-2(11bH)-yl)ethanone* (**22**)*. * Yield = 36%; ^1^H-NMR (CDCl_3_) *δ*: 2.52–2.78 (m, 3H), 2.95–3.51 (m, 6H), 3.86 (m), 4.34 (m) (total 1H), 4.15 (AB, *J* = 12.0 Hz), 4.22 (AB, *J* = 12.0 Hz) (total 2H), 4.57 (m, ), 5.10 (m) (total 1H), 7.15–7.31 (m, 4H); MS (ESI): *m/z* calcd. for [M+H]^+^: 265.1/267.1, found: 265.2/267.1.

#### 3.1.7. The Procedure for the Preparation of Compound **24**

A stirred solution of compound **23** (1.0 g, 3.1 mmol) in HCl (2 N, 10 mL) was heated to 110 °C overnight. The reaction mixture was neutralized with solid NaHCO_3_, and extracted with DCM/MeOH (V/V 5/1, 30 mL × 3). The organic phases were then processed in the usual way and chromatographed (10:1 CH_2_Cl_2_/MeOH) to afforded compound **24** (500 mg, 78%). ^1^H-NMR (CDCl_3_) *δ*: 2.80–2.88 (m, 3H), 2.95–3.01 (m, 1H), 3.54 (d, *J* = 17.6 Hz, 1H), 3.66 (m, 1H), 3.67 (d, *J* = 17.2 Hz, 1H), 4.71 (m, 1H), 5.12 (m, 1H), 6.80 (d, *J* = 5.2 Hz, 1H), 7.19 (d, *J* = 5.2 Hz, 1H); MS (ESI): *m/z* calcd. for [M+H]^+^: 209.1, found: 209.2.

#### 3.1.8. General Procedure for the Preparation of Compound **25**–**33**

To a stirred solution of compound **24** (208 mg, 1 mmol) in DCM (10 mL), cyclobutanecarbonyl chloride (136 μL, 1.2 mmol) was added and stirred at room temperature overnight. The reaction was quenched with NaHCO_3_ (aq.), extracted with DCM. The organic phases were then processed in the usual way and chromatographed (1:1 petroleum ether/EtOAc) to afforded compound **26** (150 mg, 52%). ^1^H-NMR (CDCl_3_) *δ*: 1.92–2.37 (m, 6H), 2.75 (t, *J* = 11.2 Hz, 1H), 2.84–2.96 (m, 3H), 3.27 (m, 1H), 3.75 (d, *J* = 18.8 Hz) 3.94 (d, *J* = 17.6 Hz) (total 1H), 4.18 (d, *J* = 12.8 Hz), 4.25 (d, *J* = 17.6 Hz) (total 1H), 4.71 (d, *J* = 10.4 Hz, 1H), 4.91 (d, *J* = 19.2 Hz), 5.04 (dd, *J* = 3.2 Hz, 12.8 Hz) (total 1H), 5.10 (dd, *J* = 2.8 Hz, 13.2 Hz, 1H), 6.81 (d, *J* = 5.2 Hz), 6.91 (d, *J* = 5.2 Hz) (total 1H), 7.19 (d, *J* = 4.8 Hz), 7.23 (d, *J* = 4.8 Hz) (total 1H); MS (ESI): *m/z* calcd. for [M+H]^+^: 291.1, found: 291.2.

*2-(Cyclopentanecarbonyl)-2,3,6,7-tetrahydro-1H-thieno[3',2':3,4]pyrido[1,2-a]pyrazin-4(10bH)-one* (**25**)*. * Yield = 52%; ^1^H-NMR (CDCl_3_) *δ*: 1.61–1.91 (m, 8H), 2.74 (t, *J* = 11.2 Hz, 1H), 2.86–2.97 (m, 4H), 3.76 (d, *J* = 18.4 Hz), 4.04 (d, *J* = 17.6 Hz) (total 1H), 4.50 (d, *J* = 17.6 Hz, 1H), 4.71 (d, *J* = 9.2 Hz, 1H), 4.93 (d, *J* = 19.2 Hz), 5.04 (dd, *J* = 5.2 Hz, 12.8 Hz) (total 1H), 5.15 (dd, *J* = 2.4 Hz, 13.2 Hz, 1H), 6.90 (d, *J* = 4.8 Hz, 1H), 7.19 (d, *J* = 5.2 Hz, 1H); MS (ESI): *m/z* calcd. for [M+H]^+^: 305.1, found: 305.1.

*2-(Cyclopropanecarbonyl)-2,3,6,7-tetrahydro-1H-thieno[3',2':3,4]pyrido[1,2-a]pyrazin-4(10bH)-one* (**27**)*.* Yield = 57%; ^1^H-NMR (CDCl_3_) *δ*: 1.01 (m, 2H), 1.12 (m, 2H), 1.70 (m, 1H), 2.72–2.98 (m, 4H), 4.18 (d, *J* = 17.6 Hz, 1H), 4.72 (m, 2H), 5.04–5.14 (m, 2H), 6.90 (d, *J* = 4.8 Hz, 1H), 7.19 (d, *J* = 4.8 Hz, 1H); MS (ESI): *m/z* calcd. for [M+H]^+^: 277.1, found: 276.9.

*2-Isobutyryl-2,3,6,7-tetrahydro-1H-thieno[3',2':3,4]pyrido[1,2-a]pyrazin-4(10bH)-one* (**28**)*.* Yield = 46%; ^1^H-NMR (CDCl_3_) *δ*: 1.15 (d, *J* = 6.8 Hz), 1.17 (d, *J* = 6.8 Hz), (total 6H), 2.70–2.80 (m, 1H), 2.86–2.98 (m), 3.16–3.25 (m) (4H), 3.77 (d, *J* = 18.4 Hz), 4.06 (d, *J* = 17.6 Hz) (total 1H), 4.34–4.40 (m), 4.48 (d, *J* = 17.6 Hz) (total 1H), 4.71–4.78 (m, 1H), 4.89–5.06 (m, 1H), 5.15 (d, *J* = 13.2 Hz, 1H), 6.90 (d, *J* = 5.2 Hz, 1H), 7.20 (d, *J* = 5.2 Hz, 1H); MS (ESI): *m/z* calcd. for [M+H]^+^: 279.1, found: 279.2.

*2-Pivaloyl-2,3,6,7-tetrahydro-1H-thieno[3',2':3,4]pyrido[1,2-a]pyrazin-4(10bH)-one* (**29**)*.* Yield = 76%; ^1^H-NMR (CDCl_3_) *δ*: 1.34 (s, 9H), 2.81–2.97 (m, 4H), 3.96 (d, *J* = 18.0 Hz, 1H), 4.76 (m, 1H), 4.82 (d, *J* = 17.6 Hz, 1H), 5.04(dd, *J* = 4.8 Hz, 12.4Hz, 2H), 6.87 (d, *J* = 5.2 Hz, 1H), 7.21 (d, *J* = 5.2 Hz, 1H); MS (ESI): *m/z* calcd. for [M+H]^+^: 293.1, found: 293.1[M+H]^+^.

*2-Benzoyl-2,3,6,7-tetrahydro-1H-thieno[3',2':3,4]pyrido[1,2-a]pyrazin-4(10bH)-one* (30)*.* Yield = 51%; ^1^H-NMR (CDCl_3_) *δ*: 2.86–3.02 (m, 4H), 4.08 (m, 1H), 4.36–4.38 (m, 1H), 4.88 (m, 1H), 5.01 (m, 1H), 5.19–5.25 (m, 1H), 6.98–7.62 (m, 6H), 8.10 (d, *J* = 7.6 Hz, 1H); MS (ESI): *m/z* calcd. for [M+H]^+^: 313.1, found: 313.2.

*2-(Thiophene-2-carbonyl)-2,3,6,7-tetrahydro-1H-thieno[3',2':3,4]pyrido[1,2-a]pyrazin-4(10bH)-one* (**31**)*.* Yield = 40%; ^1^H-NMR (CDCl_3_) *δ*: 2.87–2.97 (m, 4H), 4.20–4.24 (m, 1H), 4.85–5.10 (m, 4H), 6.90–7.88 (m, 5H); MS (ESI): *m/z* calcd. for [M+H]^+^: 319.1, found: 319.0.

*2-(Furan-2-carbonyl)-2,3,6,7-tetrahydro-1H-thieno[3',2':3,4]pyrido[1,2-a]pyrazin-4(10bH)-one* (**32**)*.* Yield = 46%; ^1^H-NMR (CDCl_3_) *δ*: 2.85–3.03 (m, 4H), 4.21–4.25 (m, 1H), 4.88 (m, 1H), 5.01–5.07 (m, 2H), 5.15 (m, 1H), 6.55 (d, *J* = 2.8 Hz, 1H), 6.94 (brs, 1H), 7.17 (d, *J* = 3.2 Hz, 1H), 7.22 (d, *J* = 5.2 Hz, 1H), 7.56 (s, 1H); MS (ESI): *m/z* calcd. for [M+H]^+^: 303.1, found: 303.2 [M+H]^+^.

*2-(2-Chloroacetyl)-2,3,6,7-tetrahydro-1H-thieno[3',2':3,4]pyrido[1,2-a]pyrazin-4(10bH)-one* (**33**)*.* Yield = 76%; ^1^H-NMR (CDCl_3_) *δ*: 2.82–2.98 (m), 3.24–3.29 (m) (total 4H), 3.81 (d, *J* = 18.8 Hz), 4.23 (d, *J* = 12.4 Hz) (total 1H), 4.13 (s), 4.17 (s) (total 2H), 4.38–4.46 (m, 1H), 4.75–5.06 (m, 3H), 6.87 (d, *J* = 5.2 Hz), 6.90 (d, *J* = 5.2 Hz) (total 1H), 7.22 (d, *J* = 5.2 Hz), 7.24 (d, *J* = 5.2 Hz) (total 1H); MS (ESI): *m/z* calcd. for [M+H]^+^: 285.0/287.0, found: 285.1/287.1 [M+H]^+^.

### 3.2. Killing Activity of Compounds ***1***–***33*** on S. Japonicum Adult Worms *in Vitro [36]*

Stock solutions of compounds **1**–**33** and praziquantel were prepared by dissolving 1 mg of the drugs in 0.4 ml dimethyl sulfoxide (DMSO) and adding 0.6 mL RPMI 1640 medium. *S. japonicum* worms obtained from mice (C57BL/6, female, 22–24 g, each infected with 50 cercariae) were washed in RPMI 1640 medium, kept at pH 7.5 with HEPES 20 mM and supplemented with penicillin (100 UI/mL), streptomycin (100 mg/mL) and 10% fetal bovine serum (FBS, Gibco) . After washing, 8–15 adult worms were transferred to each well of a 24-well culture plate containing 2 mL of the same medium. The worms were cultured for 30 to 60 min at 37 °C in a humid atmosphere containing 5% CO_2_, and then different concentrations of compounds **1**–**33** (10, 25, 50, 100 µM) diluted with RPMI 1640 medium were added. Control worms were treated with equal volumes of RPMI 1640 or DMSO, and worms treated with 10, 25, 50, 100 µM praziquantel were also observed. The worm mobility, tegumental alterations and parasite survival were monitored under an inverted microscope (Leica, Wetzlar, Germany) at 24, 48 and 72 h. Parasite death was defined as having no motor activity during 2 min of continuous observation as well as morphological and tegumental alterations. The tests were repeated two times when compouds showed worm killing activity below the concentration of 100 μM.

## 4. Conclusions

Given the widespread morbidity and mortality derived from schistosomiasis and the possible emergence of PZQ-resistant parasites in the near future, it is undoubtedly urgent to develop new chemotherapy for the disease. In this work, we first reinvestigated the SAR of PZQ analogues at positions 2 and 4, and we identified that the introduction of a chloroacetyl group at position 2 led to compound **7** with higher worm-killing activity against adult *S. japonicum* than PZQ *in vitro*. Second, we extended the SAR studies by replacing the aromatic moiety with a thiophene ring, which displayed similar activity as PZQ derivatives, further confirming the respective SAR. Notably, compound **33** with the chloroacetyl group killed 100% of the worms at concentrations as low as 10 μM, which represents a more potent compound than PZQ. The study of these structurally diverse compounds will likely provide meaningful information for the design of new PZQ analogues. After the initial *in vitro* screening, *in vivo* activity and toxicity will have to be tested. Design and synthesis of more potent antischistosomal agents are currently undergoing in our laboratory.
